# Microfluidic Array Chip for Parallel Detection of Waterborne Bacteria

**DOI:** 10.3390/mi10120883

**Published:** 2019-12-16

**Authors:** Lena Gorgannezhad, Kamalalayam Rajan Sreejith, Jun Zhang, Gregor Kijanka, Melody Christie, Helen Stratton, Nam-Trung Nguyen

**Affiliations:** 1 Queensland Micro- and Nanotechnology Centre, Nathan Campus, Griffith University, 170 Kessels Road, Brisbane, QLD 4111, Australia; lena.gorgannezhad@griffithuni.edu.au (L.G.); sreejith.kamalalayamrajan@griffithuni.edu.au (K.R.S.); jun.zhang@griffith.edu.au (J.Z.); h.stratton@griffith.edu.au (H.S.); 2 School of Environment and Science, Nathan Campus, Griffith University, 170 Kessels Road, Brisbane, QLD 4111, Australia; m.christie@griffith.edu.au; 3 Mater Research Institute, The University of Queensland, Woolloongabba, QLD 4102, Australia; gregor.kijanka@mater.uq.edu.au

**Keywords:** microfluidic, array, polymerase chain reaction (PCR), bacterial nucleic acids, microbial faecal source tracking (MST)

## Abstract

The polymerase chain reaction (PCR) is a robust technique used to make multiple copies of a segment of DNA. However, the available PCR platforms require elaborate and time-consuming operations or costly instruments, hindering their application. Herein, we introduce a sandwiched glass–polydimethylsiloxane (PDMS)–glass microchip containing an array of reactors for the real-time PCR-based detection of multiple waterborne bacteria. The PCR solution was loaded into the array of reactors in a single step utilising capillary filling, eliminating the need for pumps, valves, and liquid handling instruments. Issues of generating and trapping bubbles during the loading chip step were addressed by creating smooth internal reactor surfaces. Triton X-100 was used to enhance PCR compatibility in the chip by minimising the nonspecific adsorption of enzymes. A custom-made real-time PCR instrument was also fabricated to provide thermal cycling to the array chip. The microfluidic device was successfully demonstrated for microbial faecal source tracking (MST) in water.

## 1. Introduction

The faecal pollution of water is one of the major threats to human health. Understanding the origin of faecal pollution is necessary for identifying public health risks, devising effective management, and preventing further pollution [1]. To date, water resource managers responsible for monitoring microbiological water quality have a limited number of conventional tools available. Cultivation-based approaches for standard faecal indicator bacteria enumeration (SFIB) [2] are time-consuming and not informative about pollution sources.

To overcome the drawbacks of the traditional SFIB method, molecular microbial source tracking (MST) approaches have been developed over the last few years and can link faecal pollution to its origin [3,4]. The polymerase chain reaction (PCR) and quantitative PCR (qPCR) have been widely used as tools for specific nucleic acid-based tests [5,6], particularly for the detection and quantification of source-associated genetic markers in water samples [7]. Over the years, multiplex PCRs and microarrays have been introduced as the two powerful high-throughput genomic technologies [8]. Simultaneous detections of multiple waterborne bacterial pathogens have been reported employing a multiplex PCR system [9]. However, multiplex PCRs requires accurate primer designs and an optimal reaction mixture to avoid the probability of primer dimer formation that may result in the preferential amplification of certain targets [10]. Microarrays are another alternative method for analysing the pathogen content in wastewater. Microarrays provide quick detection of multiple target genes of multiple organisms simultaneously due to its ability of screening a large number of sequences [11]. Even though microarrays have been broadly used for simultaneous detection, there are still limitations hindering their applications such as a low sensitivity and variability of the result as compared to those of real-time PCRs [12].

Platforms capable of integrating PCRs with microarray techniques have been introduced as a probable solution to alleviate the existing difficulties in the application of microarrays. Some of these systems integrate solid-phase PCRs with microarrays. However, recent studies have indicated that the solid-phase PCR efficiency is less than that of the solution-phase PCR [13].

Recent advances in microfluidics have enabled the development of new cost-effective array devices for the multiplex detection of contaminants. The main advantages of microfluidics-based array platforms are the lower cost of purchase and operation, fast analysis, high sensitivity, and minimum infrastructure requirements [14]. However, there are still some bottlenecks in the design and operation of these systems. Most PCR-based microfluidic devices need to load the PCR mixture into reaction wells using costly liquid-dispensing robots or pneumatic pressure sources, or the immobilization of primers in a solid matrix [15,16,17].

The development of manual liquid loading methods without handing robots or external pumps is challenging for microfluidic arrays. The manual liquid loading process comprises nucleic acid sample loading into the reaction wells, followed by the isolation of these wells to prevent cross-contamination.

Recently, a few research teams have successfully used micropumps in conjunction with an array of valves for performing PCRs on microfluidic chips. Liu et al. [17] developed a microfluidic device that integrates thousands of hydraulic valves and pneumatic pumps, allowing the distribution of 2 µL of PCR mixture among 400 independent reactors. The implementation of valves and pumps lead to complex fabrication and operation processes and may also increase the size of the chip.

The sealing of a microfluidic array is another significant challenge that needs to be addressed. Based on the materials that are employed in chip fabrication, sealing methods require specific equipment and protocols. Polydimethylsiloxane (PDMS), pressure-sensitive adhesive foil, adhesive sealing film, and ultraviolet (UV) adhesive are the most commonly used materials for sealing devices for the PCR [7]. In addition, a microPCR array chip with open reactors has also been reported [18,19].

In this context, we present a simple and inexpensive microfluidic PCR array. We used a glass–PDMS–glass configuration to reduce water loss during PCR thermal cycling. By the robust surface modification of the PDMS, the absorption of proteins/nucleic acids on the surface of this material was limited. Array reactors were preloaded with primer pairs. The loading process only required two pipetting steps to introduce the sample and oil. Sealant was used to enclose sample loading/air venting ports to prevent liquid movement and evaporation. The dimensions of the sealant were 5 cm length, 1 cm width, and 1 mm height. There was no need for additional components or external equipment for the operation. Finally, the capability of the array chip was verified by the simultaneous detection of three human-associated MST markers: *Escherichia coli* (*E.coli*) (H8), human-specific *bacteroidals* (Gen bac III), *E.coli* (UidA).

## 2. Materials and Methods

### 2.1. Chip Design

**Figure 1:**
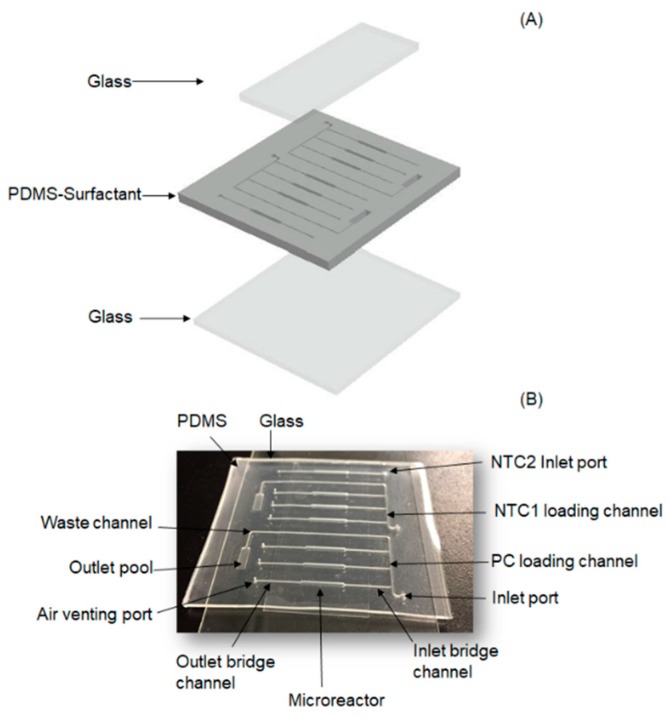
Microfluidic polymerase chain reaction (PCR) chip: (**A**) three layers of the device; (**B**) fabricated PCR chip.

Figure 1 shows the proposed microfluidic PCR chip. The device was designed as an array for the parallel detection of three human wastewater-associated MST markers. The array chip, including microreactors, sample loading ports and waste channels, inlet and outlet bridge channels, and air venting ports, was made of polydimethylsiloxane (PDMS) and glass. A non-ionic surfactant, Triton X-100 (TX-100) (Sigma Aldrich, St. Louis, MO, USA), was used as a surface modifier. The surfactant provides a great reduction in contact angle and prevents the absorption of macromolecules onto the PDMS surface [20]. Contact angle measurements and UV-Vis studies were carried out to evaluate the effect of TX-100 on the wettability changes of the pure PDMS (Figures S1 and S2). Over the chip fabrication step, different microreactors were loaded with dry primer pairs for the simultaneous detection of targeted MST markers.

The loading channel was employed for distributing the PCR mixture containing DNA templates into the array of microreactors. Loading the PCR mixture into microreactors purges out the air inside the device through the air venting ports, facilitating liquid flow. After filling the microreactors, the extra liquid samples inside the microreactors were isolated from each other. The inlet and air venting ports were sealed with a sterile mineral oil and a commercially available sealant to avoid sample movement and evaporation during the PCR thermal cycling process. The smooth internal surface of the microreactors restricts the generation and trapping of air bubbles during sample loading and PCR thermal cycling. Scanning electron microscopy (SEM) results are shown in Figure S3.

The existence of air bubbles inside the microreactors pushes the liquid out due to bubble expansion. Furthermore, air bubbles also have a negative effect on PCR efficiency by creating various temperature zones in microreactors. Figure 1B shows the image of the microfluidic array chip, including an array of seven microreactors for the PCR. The chip contains three microreactors connected to a common loading channel for the target sample (positive control reaction; PC), three microreactors connected to another common loading channel for a no-template control reaction in the presence of a wrong target (NTC1), and a single microreactor for a no-template control reaction with water (NTC2). Typical dimensions of the array chip are as follows. The bridging channels are 10 mm long, 300 µm wide, and 500 µm high. The microreactors are 10 mm long, 1 mm wide, and 500 µm high, resulting in a volume of 5 µL. Reactors 1–3 were pre-loaded with dried primer pairs, which are specific for EC H8, Gen bac III, and UidA, respectively. If a PCR mixture containing a bulk of standard DNA templates (for *E.coli* H8, Gen bac III, and UidA) was loaded into the array, the mixture would fill the microreactors. During the amplification process, the primer pairs specifically hybridised to their desired targets. Reactors 4–6 were also pre-loaded with dried primer pairs (similar to reactors 1–3), to perform the NTC1 for the corresponding reactions. For NTC1, the PCR mixture without the standard DNA template, which contains a DNA template from Staphylococcus aureus, was loaded into the NTC1 loading port. Reactor 7 was also pre-loaded with one dried set of primer pairs to perform the NTC2. For NTC2, the PCR mixture containing just water was loaded into the array to check the possible contamination in that.

### 2.2. Chip Fabrication

The microfluidic array chip described above is a glass–PDMS–glass sandwich configuration. Figure 1A schematically illustrates the fabrication process of the array chip. AutoCAD (Autodesk Inc., San Rafael, CA, USA) was used to design the mould for making the PDMS layer. The PMMA module was prepared by the micro-milling method (LPKF Laster& Electronics Proto Mat S 43 at Nanjing University of Science and Technology, Nanjing, China). After the completion of the module, a PDMS pre-polymer (Dow Corning Sylgard 184, Dow Corning, Midland, MI, USA) and a cross-linker were mixed in a 10:1 ratio by weight [21]. Subsequently, Triton X-100 (Sigma Aldrich, St. Louis, MO, USA) at a 0.5% weight percentage was added to liquid PDMS [20]. Later, the mixture was degassed in vacuum for around 20 min. Subsequently, the mixture was poured over the mould with a thickness of 1 mm and degassed in vacuum for 10 min and cured at 67 °C for 3 h. After being completely cured, the PDMS was peeled off the mould, and the venting ports were punched. The resulting PDMS layer was washed with acetone, isopropanol, and milliQ water and dried at 67 °C for 30 min. Another PDMS mixture with a pre-polymer/cross-linker ratio of 10:1 by weight was spread on two acetone-washed glass slides and then bonded to the PDMS layer. Finally, the device was cured at 67 °C for 48 h to enhance the bond between the PDMS part and the glass layers. The mixtures of forward and reverse primers for each marker were loaded to the preferred microreactor by pipetting through the air venting ports. The primers were then dried by annealing the chip at 67 °C for 10 min.

### 2.3. Chip Operation

**Figure 2:**
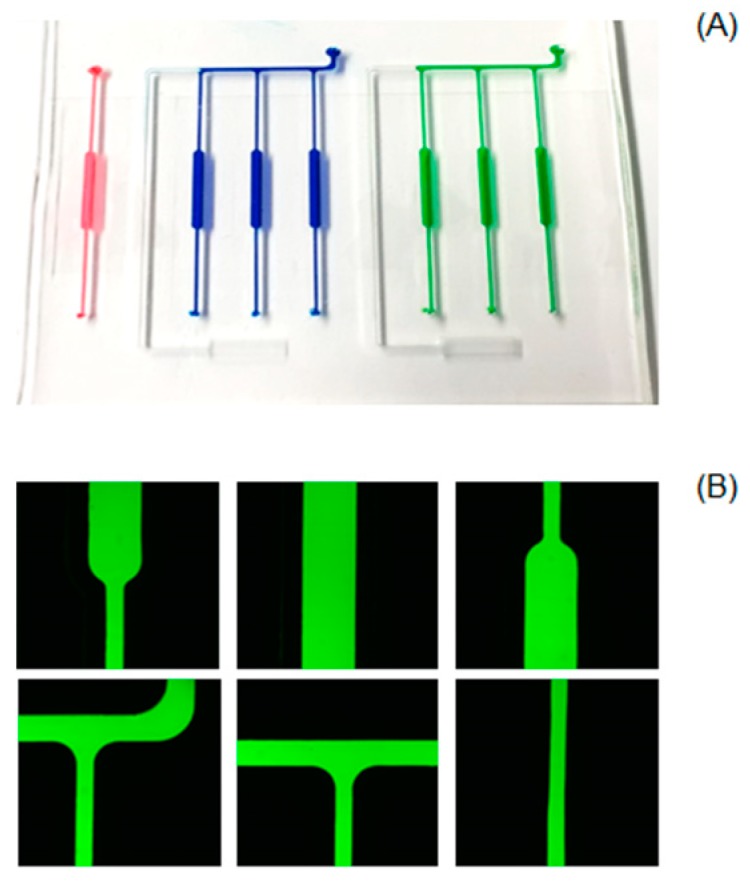
Liquid sample loading and isolation of the reactors. (**A**) Test with food colouring; (**B**) test with fluorescent dye.

Figure 2 shows the basic operation procedure of the chip. The hydrophobic nature of PDMS avoids the liquid solutions flowing easily into the microreactors through capillary filling. The addition of 0.5% of Triton X-100 into PDMS resolved this problem. The resulting reduction in contact angle allows the PCR mixture to flow into the reactors and channels by capillary action [20]. The loading process does not need an external pump. After a single step of manual pipetting of the PCR solution into the loading port, the capillary force completed the fluidic operation process, and the extra PCR mixture was collected in the outlet pool. The sealing performance of the chip was checked using food colouring. Figure 2 shows that the reactors were well sealed and isolated from each other: POC (blue), NTC1 (red), and NTC2 (green).

The smooth inner surfaces of the microreactors prevented bubbles from forming and being trapped during sample loading. Trapped bubbles are one of the key challenges in designing a PCR chip [22]. This smoothness was the result of the microreactor design with curved corners and the use of liquid PDMS prepolymer as an adhesive material to bond the cured PDMS layer and glasses. The absence of bubbles during the loading step was clearly confirmed by a fluorescent dye test, Figure 2B.

**Figure 3:**
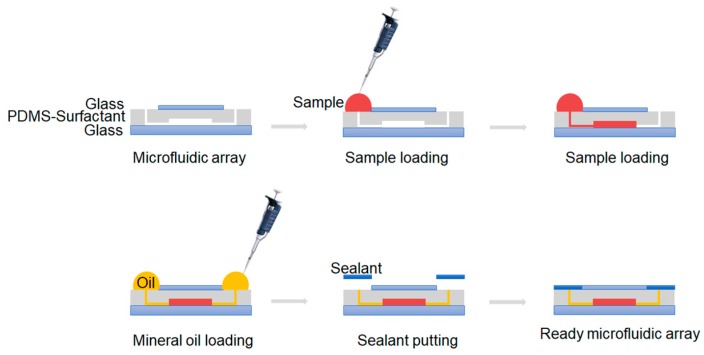
Cross-sectional views of chip operation.

After filling the microreactors, sterile mineral oil was added into the input and gas venting ports to hold the sample in microchambers and to avoid their movements during thermal cycling. The oil also reduced the evaporative loss of the sample. Finally, a commercially available sealant was used to completely seal the ports. Figure 3 schematically outlines the operating procedure of the microdevice.

### 2.4. Real-Time Polymerase Chain Reaction (PCR) Instrument

**Figure 4:**
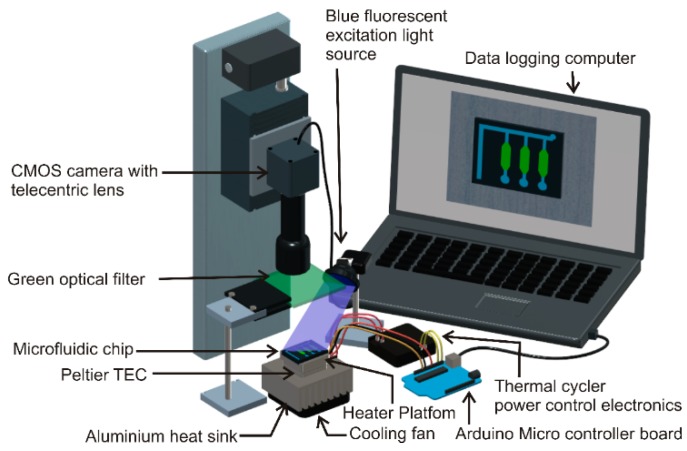
Experimental setup of the PCR thermal cycling platform.

A customised thermal cycler was developed to run the PCR thermal cycles on the proposed microfluidic chip. A 5 × 5 × 2 cm^3^ aluminium block embedded with a 60 W cartridge heater (Core electronics, Kotara, Australia) was used as the thermal cycling platform. A LM35 temperature sensor (Core electronics, Kotara, Australia) was attached to the aluminium block using heat-conductive glue to monitor the temperature. The thermal cycling platform was mounted on a Peltier thermoelectric cooler (TEC-12706, Aus electronics, Chipping Norton, Australia), which was, in turn, attached to a heat sink-cooling fan assembly, as shown in Figure 4. The temperature of the platform was controlled by a proportional-integral-derivative (PID) algorithm implemented in an Arduino UNO microcontroller board. The thermal cycler was programmed to run the following temperature cycles: Initial denaturation at 95 °C for 45 s followed by 35 cycles of denaturation at 95 °C for 15 s and annealing at 60 °C for 1 min. The thermal ramping rate of the custom-made thermal cycler was 0.20 K/s during heating and 0.26 K/s during cooling. The temperature difference between the aluminium block and the sample inside the chip was measured using a pre-calibrated negative-temperature-coefficient thermistor (Figure S4A,B).

### 2.5. Bacterial Samples and PCR Experiment

Thirty millilitres of faecal slurry from the mixed human samples was spun at 4 °C for ten minutes at 5000 rpm to concentrate the sample. The total DNA was extracted from the pellet after mixing with 10 parts ASL buffer using the QIAamp DNA stool mini kit according to the instructions of the manufacturer (QIAGEN, Victoria, Australia). The resulting DNA extracts were stored at −20 °C until actual use. The resulting DNA was used as the template DNA to synthesise the standard DNA for desired gene sequences. First, the concentration of DNA was estimated by a nanodrop instrument (BioLab, Ipswich, MA, USA) and diluted to reach a concentration of around 100 ng/µL. Next, one PCR was run for each set of primers in the presence of the template DNA. The sequences of forward and reverse primers are listed in Table 1. The PCR solution (20 µL) contained 2.5 µL forward primer, 2.5 µL reverse primer, 2.5 µL DI water, 2.5 µL template DNA, and 10 µL Gotaq^®^ green master mix (Promega, Madison, WI, USA). The mixture was placed in a conventional PCR instrument (Biorad CFX Connect, BioRad, Hercules, CA, USA) with the thermal cycling condition of initial denaturation at 95 °C for 45 s followed by 35 cycles of denaturation at 95 °C for 15 s and annealing at 60 °C for 1 min. The resulting amplicons, which were synthetic DNA for the desired gene, were then ready to be used for on-chip amplification. Three synthetic DNA samples were mixed together to prepare a usable template DNA for on-chip amplification. The bulk of DNA plus water and SsoFast EvaGreen supermixes (Bio-Rad, USA) was then added to the chip, which had the primer pairs immobilised in all reactors beforehand. Next, the chip was placed on top of a custom-made thermal cycler to tolerate the thermal cycling condition. The results were verified via both fluorescence measurement and agarose gel (1.5%) electrophoresis followed by exposure under a UV transilluminator (Bio-Rad).

## 3. Results

### 3.1. Simultaneous Detection of Microbial Faecal Source Tracking (MST) Markers Using Microfluidic Device

The two bacteria of *Escherichia coli* (*E. coli)* and *Bacteroidales* were selected to demonstrate the function of the chip. *E. coli* and *Bacteroidales* are gram-negative bacteria flourished in the intestine with properties that both positively and negatively affect the host [23]. Infection with some species of this bacteria, either untreated or with delayed treatment, can lead to infections in the host [24,25]. The high level of *E.coli* and *bacteroidales* in the gut of humans and animals has made them two of the most important faecal indicator bacteria in microbial source tracking (MST) assays in waters. We selected two target genes for *E. coli* (UidA, H8) and one target gene for *Bacteroidales* (Gen bac III) to demonstrate the function of the chip. The performance of the chip was examined by detecting sequences of Gen bac III, UidA, and H8 in a bulk of standard DNA extracted from *Bacteroidales* and *E. coli*. The microdevice was loaded with PCR solution containing a predefined concentration of DNA templates. The temperature of the chip was controlled by the custom-built portable and programmable PCR apparatus described in the previous section. The numerical values of fluorescent intensities of the amplicons at various time intervals were captured by a camera and evaluated using ImageJ (National Institutes of Health, Bethesda, MD, USA) [26], an open-source software. Next, the fluorescent intensity values of the samples inside the microchambers of the device were normalised. Normalisation of the fluorescent intensities was achieved by the following formula [27]:

I*_st_ = (*I*_st_ − *I*_s0_)/*I*_max_, (1)

where *I*_st_ is the fluorescent intensity of the sample in each microchamber measured at a given time, *I*_s0_ is the fluorescent intensity of that sample at the start of thermal cycling, and *I*_max_ is the maximum fluorescent intensity measured among all the samples.

**Figure 5:**
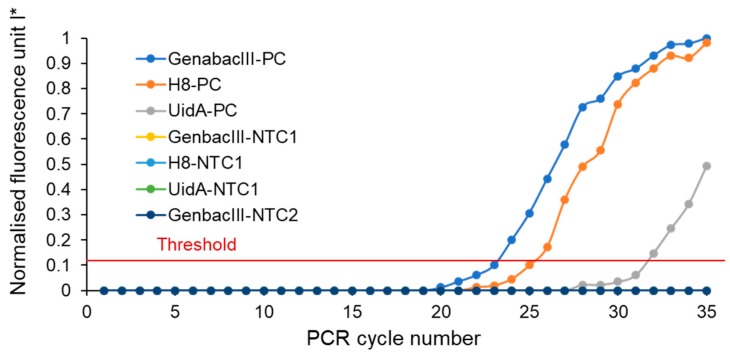
Amplification plot for on-chip detection of *Bacteroidales* and *Escherichia coli (E. coli)*. Positive controls (PC): Bulk of standard templates of DNA containing GenbacIII (71,800 copies/µL), H8 (52,300 copies/µL), and UidA (13600 copies/µL) sequences. Negative controls (NTC): NTC1 (template DNA from Staphylococcus aureus), NTC2 (no template DNA; water).

Figure 5 presents the amplification curve from microreactors on the device. The normalised fluorescent intensity increases with increasing cycle number in all microchambers containing positive controls (PC). The increase in fluorescent intensity of the PCR solution indicates a positive polymerase chain reaction within the microreactors. The microreactors containing negative controls of the PCR mixture, for the wrong target (NTC1) and for water (NTC2), show no significant fluorescence over thermal cycling. This demonstrates the specificity of the PCR inside the microreactors. Maximum fluorescence was observed in the microreactor containing primer pairs for detecting the Gen bacIII gene. The reason can be attributed to the high concentration of this sequence (71,800 copies/µL) in the bulk of the template DNA sample. The level of maximum fluorescence signal was less in microreactors with H8 and Uid A primer pairs due to the smaller amount of DNA copies of 52,300 and 13,600 copies/µL, respectively.

The threshold line is the maximum level of fluorescence that can be defined as background. The first cycles of the run show the initiation phase. In this phase, all PCR mixture components were in abundance, but the number of amplicons present in each reaction was negligible to produce a fluorescent signal that exceeds the threshold line. By progressing the experiment, the amount of fluorescence signal will exceed the threshold line. The cycle threshold (*C*_t_) is the cycle number at which the fluorescent signal reaches the threshold line [28]. The values of *C*_t_ for the PCR with primer pairs of Gen bacIII, H8, and UidA were recorded as 23, 25.2, and 31.8, respectively.

### 3.2. Performance Characteristics of the Developed Microfluidic Device

**Figure 6:**
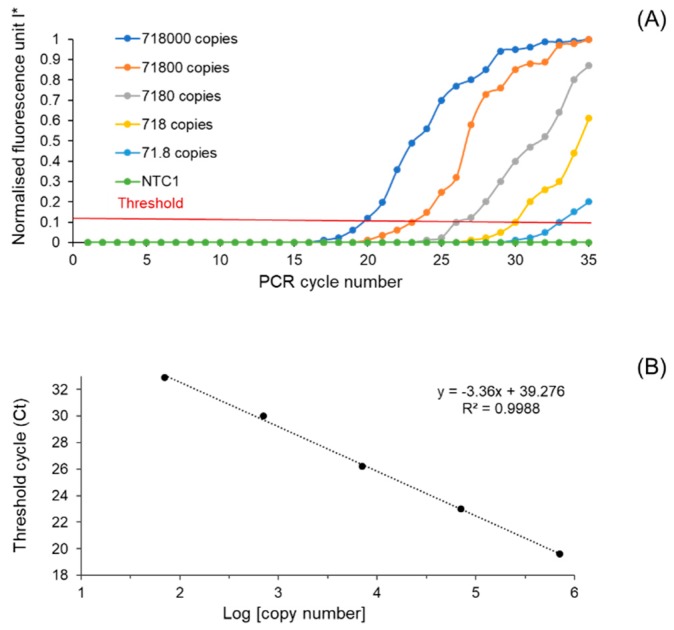
(**A**) Amplification plot for 10-fold serially diluted standard template DNA (Gen bac III sequence); NTC1 (template DNA from Staphylococcus aureus). (**B**) Standard curve for 10-fold serially diluted standard template DNA.

We measured the limit of detection (LOD) to examine the performance of the microdevice on our real-time PCR instrument. The LOD measurement was performed by the DNA dilution series of the Gen bac III sequence ranging from 718,000 to 71.8 DNA copies/μL. In this experiment, the microreactors were preloaded with Gen bac III primer pairs. Each concentration was then added separately to the specific channel through the air venting ports. Figure 6 shows the on-chip real-time PCR of the serially diluted Gen bac III sequence carried out on our customised thermal cycler instrument.

We observed that the trends in fluorescent intensities in all concentrations increased with increasing PCR cycles. In comparison, no signal was detected in the negative target control (NTC1). The LOD was estimated to be 71.8 copies (Figure 6A). The regression equation was calculated to be *y* (threshold cycle) = −3.36 (log of copy numbers) + 39.276, with a correlation coefficient (*R*^2^) of 0.99 (Figure 6B). The PCR efficiency of the primer pair was 98.44%, which was calculated using the following equation:

*E* = 10 ^(−1/slope)^ − 1. (2)

These high levels of *R*^2^ and the amplification efficiency value confirm the successful PCR and amplification of the template DNA in our platform. The LOD of our method is comparable to some microfluidic array systems reported previously [29]. Although some other studies have reported better LODs, their method required more complicated and expensive chip fabrication processes [30,31].

### 3.3. Validation of Real-Time PCR Using Gel Electrophoresis

**Figure 7:**
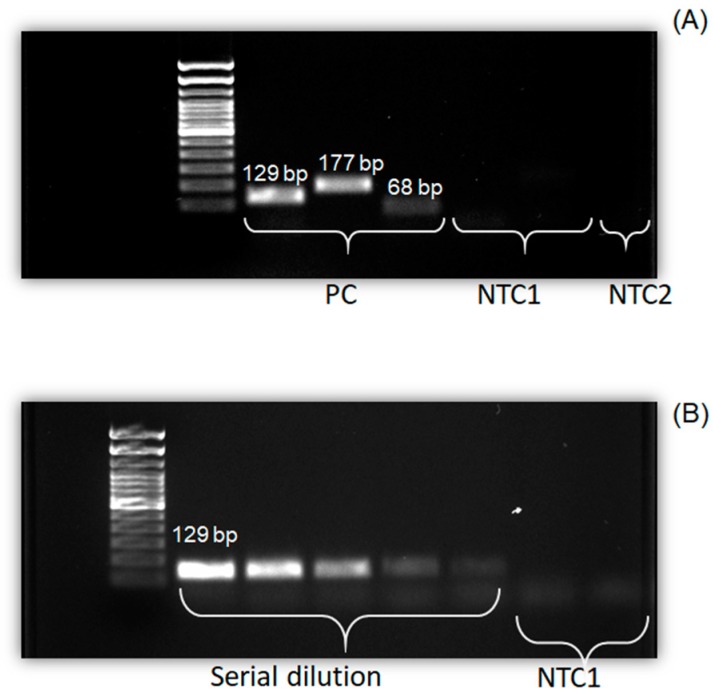
Gel image of on-chip PCR products. (**A**) Illustration of GenbacIII, H8, UidA, and negative controls (NTC1, NTC2). (**B**) Image of serial dilution of GenbacIII sequence.

To validate our optical measurements in another platform, we used gel electrophoresis for on-chip amplicons analysis. In this experiment, the resulting amplicons after PCRs were pipetted out through the air venting ports and loaded on a gel which was preloaded with a ladder (100–20,000 bp). Figure 7A shows the result of the simultaneous amplification of standard templates of DNA containing GenbacIII (71,800 copies/µL), H8 (52,300 copies /µL), and UidA (13,600 copies/µL) sequences on the developed microdevice. The banding pattern analysis on the gel revealed three distinct bands of 129, 177, and 68 bp for GenbacIII, H8, and UidA, respectively. These bands were reported before using some off-chip methods [32,33,34]. The appearance of no significant bonds in the negative controls for untargeted samples such as *Staphylococcus aureus* and water (NTC1 and NTC2) demonstrates the specificity of our platform.

Figure 7B presents the resulting amplified amplicons of the serially diluted GenbacIII sequence. The band intensities gradually decrease for lanes from left to right, as the amounts of template DNA (copy numbers) were reduced in the PCR mixture. No significant change was observed for NTC1.

## 4. Conclusions

We designed and developed a real-time and quantitative PCR system for nucleic acid detection. The system is based on a disposable chip specifically designed to be compatible with our custom-made thermal cycling and optical detection system. The advantages of our chip are simple preparation and application, low cost, parallel detection of various marker genes, capillary flow-based sample loading, and no need for external pumps or valves. Evaporative loss and PCR efficiency, which are two significant technical challenges in designing the chips, were successfully addressed. We used glass on the top and at the bottom of the PDMS layer, and oil on both sides of the microreactors, to capture the PCR sample and prevent its movement. Furthermore, we used sealant to close the input/air venting ports. Bubble generation was another challenge in chip design and can decrease the PCR efficiency. We solved this issue with bubbles by employing rounded corners and a smooth internal surface for the microreactors. We also demonstrated that the surface modification of PDMS using a surfactant (Triton X-100) can effectively prevent undesired protein adsorption and subsequently improve PCR amplification. The function of the chip was verified with successful microbial source tracking for three human faecal markers. Future works would be extended to develop chips with other materials to facilitate chip sealing or even omit the sealing step.

## Figures and Tables

**Table 1:** Sequences of forward and reverse primers for the desired target organisms.

Target Organism	Primer	Sequence (5ʹ-3ʹ)	PCR Product Size (bp)
*Bacteroidales*	GenbacIII-F GenbacIII-R	GGGGTTCTGAGAGGAAGGT CCGTCATCCTTCACGCTACT	129
*Escherichia coli* *(E. coli)*	H8-F H8-R	ACAGTCAGCGAGATTCTTC GAACGTCAGCACCACCAA	177
*E. coli*	UidA-F UidA-R	CGGAAGCAACGCGTAAACTC GAGCGTCGCAGAACATTACATT	68

Ethical approval for the study of the effectiveness of molecular assays in detecting human faecal pollution (4 February 2013), and the effect of freezer storage on human faecal samples was done by the ethics committee through Griffith University office of research (Reference No. BPS/01/13/HREC, 4 February 2013). Written informed consent was obtained from all participants.

## Supplementary Materials

The following are available online at https://www.mdpi.com/2072-666X/10/12/883/s1, Figure S1: Contact angle evaluation of the chips; Figure S2. UV-Vis spectra of the chips; Figure S3. Scanning electron microscopy (SEM) images of a channel inside the chip; Figure S4. Comparison of the aluminium block temperature and the sample inside the chip: (**A**) The experimental setup; (**B**) Measured temperatures of the heater block and the microfluidic device.
