# Supplementary material for: Microfluidic Array Chip for Parallel Detection of Waterborne Bacteria
Source: Micromachines (Basel). 2019 Dec 16;10(12):883. doi: 10.3390/mi10120883 (PMC6952809; doi:10.3390/mi10120883)
Supplement: Supplementary file 1 [file micromachines-10-00883-s001.pdf]

micromachines

Supplementary Materials

Microfluidic Array Chip for Parallel Detection of Waterborne Bacteria

Lena Gorgannezhad, Kamalalayam Rajan Sreejith, Jun Zhang, Gregor Kijanka, Melody Christie, Helen Stratton and Nam-Trung Nguyen

S1. Contact angle measurements were carried out to analyse the wettability changes of the chip surface (Figure S1). The fabricated chips using pure polydimethylsiloxane (PDMS) and modified PDMS (0.5% TX-100) were characterised. The effect of the washing step on surface characterisation was also investigated. A droplet of water was placed on the chip and images of the contact angle were captured at 10 min intervals. The images were then evaluated using imageJ (software). The results demonstrated that the initial contact angles (time = 0 min) for pure PDMS and washed PDMS + Surfactant were very close, 117° and 115.8°, respectively. However, unwashed PDMS + Surfactant showed a lower contact angle of 113° due to the excess amount of surfactant. The time-dependent wettability changed the surfaces on which the experiment was performed in a 10 min duration. Within several minutes, the contact angles of all surfaces started to decrease. However, the decrease in contact angle for washed PDMS + Surfactant was more than that of pure PDMS. Unwashed PDMS + Surfactant showed a final contact angle of 72.8°.

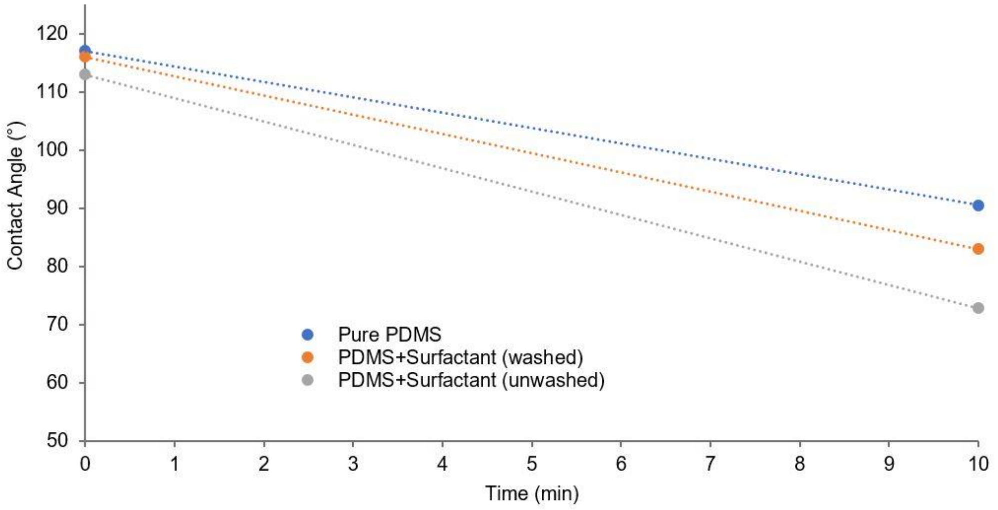

Figure S1. Contact angle evaluation of the chips.

S2. The UV-Vis study was performed to optically test the transparency of the chips. The optical property of pure/modified PDMS was characterised by measuring the transmittance with a UV/Vis spectrophotometer. The spectra were obtained for samples over a wavelength range of 200–700 nm. The result showed that the transmittance of the pure PDMS decreased by adding 0.5% surfactant. However, based on our real-time experimental results and a published study [1], this surface modification had a negligible effect on fluorescence transmission. In addition, washing the chip had no significant impact on the transparency of the PDMS + Surfactant.

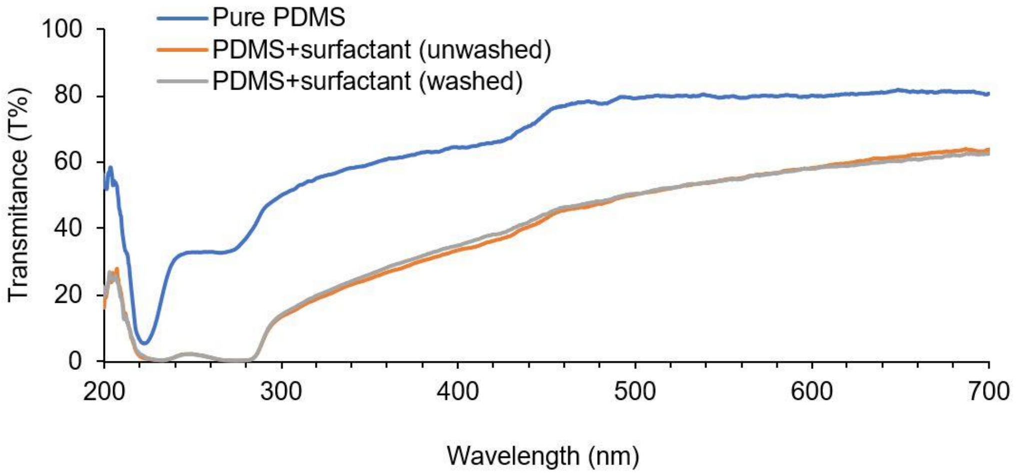

Figure S2. UV-Vis spectra of the chips.

S3. The surface morphology of the chip was investigated by scanning electron microscopy (SEM) using a JSM-6510LV with an acceleration voltage of 15 kV. It can be found that the edges and surface were smooth enough to prevent bubble generation during loading.

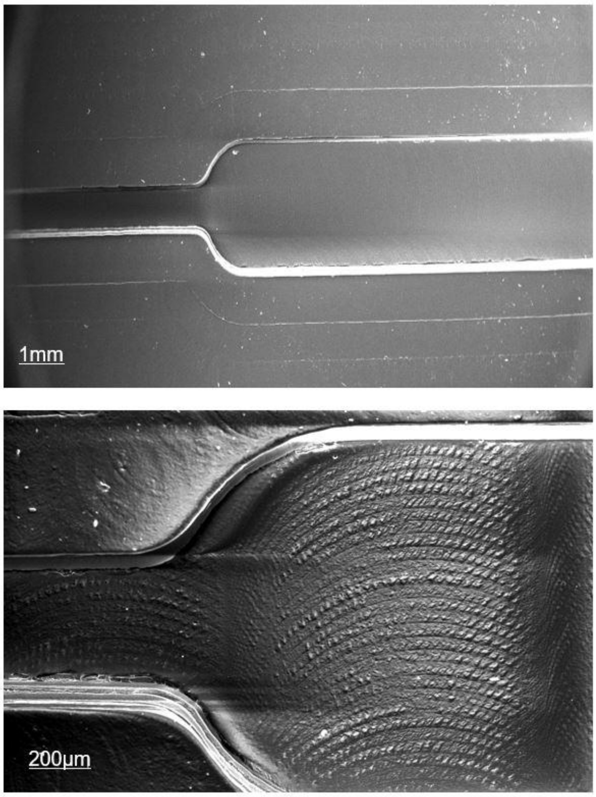

Figure S3. Scanning electron microscopy (SEM) images of a channel inside the chip.

S4. The temperature difference between the aluminium block and the sample inside the chip was measured. A pre-calibrated negative-temperature-coefficient (NTC) thermistor (Build Circuit, Australia) was inserted between the PDMS upper layer and the glass bottom layer of a microfluidic chip similar to the one explained in the manuscript, as shown in the Figure S4A. A small incision was made on the top PDMS layer of the microfluidic chip to insert the NTC thermistor. This incision was

later closed using a UV curing polymer, and the thermistor was placed intact. It has to be noted that it is not possible to put any thermally conductive paste between the thermistor and sandwiching layers of PDMS (on top) and glass (at the bottom) in this arrangement. Thus, the heat transfer efficiency to the sensor might be a little less than the actual scenario, and the temperature reading inside the chip might be a little less than the actual values. The microcontroller was programmed to heat up the aluminium block to a constant temperature, and the corresponding temperature inside the microfluidic chip was measured using the thermistor. The results of the experiments are depicted below (Figure S4B). The temperature readings from the inside part of the microfluidic chip is satisfactory considering the minor heat transfer inefficiency of the measurement setup. The average temperature difference between the aluminium block and the inside of the microfluidic chip was observed to be 2.3 ± 0.8 K over the temperature ranges under consideration. The reading provides evidence for the satisfactory heat transfer between the aluminium block and the PCR sample inside the microfluidic chip.

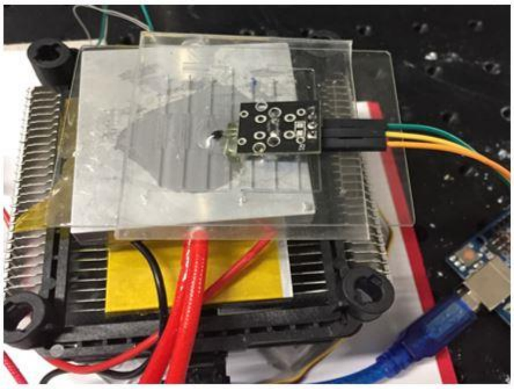

(A)

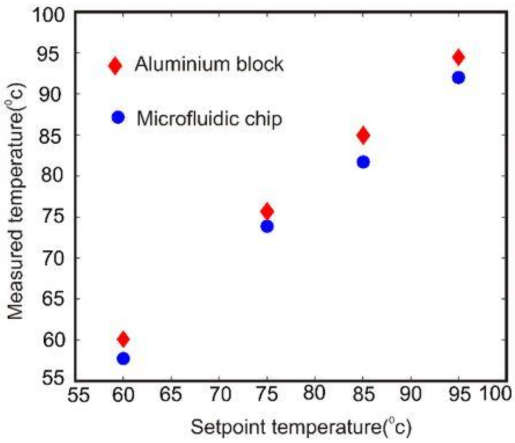

(B)

Figure S4. Comparison of the aluminium block temperature and the sample inside the chip. (**A**) The experimental setup; (**B**) Measured temperatures of the heater block and the microfluidic device.

References

1. Fu, Y.; Zhou, H.; Jia, C.; Jing, F.; Jin, Q.; Zhao, J.; Li, G. A microfluidic chip based on surfactant-doped polydimethylsiloxane (PDMS) in a sandwich configuration for low-cost and robust digital PCR. *Sens. Actuators B* **2017**, *245*, 414–422.

© 2019 by the authors. Submitted for possible open access publication under the terms and conditions of the Creative Commons Attribution (CC BY) license (http://creativecommons.org/licenses/by/4.0/).
